# Refractory immune thrombocytopenia successfully treated with high-dose vitamin D supplementation and hydroxychloroquine: two case reports

**DOI:** 10.1186/1752-1947-7-91

**Published:** 2013-04-04

**Authors:** Barry Bockow, Tamara Bockow Kaplan

**Affiliations:** 1 Arthritis Northwest, 16122 8th Ave SW, Seattle, WA, 98166, USA; 2 Department of Medicine, University of Washington School of Medicine, 1959 N.E. Pacific Street, Seattle, WA, 98195, USA; 3 Department of Medicine, Brigham and Women’s Hospital, Harvard Medical School, 75 Francis Street, Boston, MA, 02115, USA

**Keywords:** Immune thrombocytopenia, Plaquenil®, Platelets, Prednisone, T regulatory cells, Vitamin D

## Abstract

**Introduction:**

Immune thrombocytopenic purpura is thought to be characterized by an immune response against the host’s own platelets. If the thrombocytopenia is severe, patients are initially treated with high-dose steroids. Other more toxic second line treatments are considered if steroids fail. Here, we report the case of two patients in whom conventional treatment was unsuccessful but who responded to hydroxychloroquine and high-dose vitamin D replacement therapy. To the best of our knowledge, this is the first description of successful treatment for immune thrombocytopenia with high-dose vitamin D and hydroxychloroquine.

**Case presentation:**

Case 1: We report the case of a 79-year-old Caucasian man who presented with high titer antinuclear antibodies, positive anti-SSA/Ro autoantibodies and clinically was felt to have an overlap of systemic lupus erythematosus and/or Sjögren’s syndrome with profound life-threatening thrombocytopenia. There was no evidence of underlying malignancy. The patient’s platelet count significantly increased with vitamin D and hydroxychloroquine treatment, but upon vitamin D discontinuation his platelet levels plummeted. Hydroxychloroquine therapy was maintained throughout treatment. With reinstitution of high-dose vitamin D therapy, platelet counts were restored to normal levels.

Case 2: We also report the case of an 87-year-old Caucasian woman who presented with high titer antinuclear antibodies, positive anti-SSA/Ro autoantibodies and was felt to have an overlap of systemic lupus erythematosus and/or Sjögren’s syndrome with immune thrombocytopenia; she also had severely low levels of 25-hydroxy vitamin D (17ng/mL). There was no evidence of underlying malignancy. She responded to high-dose vitamin D replacement and hydroxychloroquine treatment, thereby alleviating the need for high-dose steroid treatment. She remains in remission while taking vitamin D, hydroxychloroquine and very low-dose prednisone. No untoward side effects were observed in either patient.

**Conclusions:**

In our two case reports, we found an association between vitamin D deficiency and immune thrombocytopenia where platelet levels responded to vitamin D treatment and hydroxychloroquine but not to prednisone. We believe there may be synergism between vitamin D supplementation and hydroxychloroquine. The mechanism by which high-dose vitamin D results in increased platelet counts in immune thrombocytopenia patients is unknown. However, vitamin D has long been thought to play an immunomodulatory role, which may include a dampened immune response in patients with immune thrombocytopenia or other autoimmune diseases.

## Introduction

Immune thrombocytopenic purpura (ITP) is thought to be an autoimmune disorder mediated by autoantibodies with no known etiology, and the incidence is approximately 2.5 per 100,000 persons per year [1]. The goal of treatment is to keep the platelet count above 3×10^4^/mm^3^ to prevent major internal organ bleeding.

Current treatment involves intravenous corticosteroids, immunosuppressants such as mycophenolate mofetil and azathioprine, Cytoxan® (cyclophosphamide), and intravenous immunoglobulin (IVIg) [2]. Anti-D immunoglobulin can only be given to RhD-positive individuals, thus limiting treatment options for RhD-negative persons [3]. Rituximab, a chimeric monoclonal antibody, has also been tried as an experimental treatment [4], and combinations of therapies have been used with some success [5]. Each of these therapies may be associated with significant side effects and efficacy is often temporary, requiring either additional and/or alternative treatment. Many of the side effects of steroids are well known and include hypertension, diabetes, osteoporosis and adrenal insufficiency. Several of these drugs are carcinogenic and there have been reports of malignancy formation after treatment, for example a high risk of acute myelogenous leukemia after Cytoxan® treatment [6]. Response rates vary, and there is currently no consensus regarding the appropriate treatment protocol for this condition [7]. Corticosteroids show efficacy in 50% to 80% of cases, but if treatment is stopped, the remission rate is only 10% to 30% [8]. If patients are refractory to drug treatments, splenectomy is a second line option; two-thirds of patients who undergo splenectomy for ITP respond to the treatment [9]. However, complications may arise from this surgical procedure including hemorrhage, abscess, sepsis, thrombosis and death and relapse of ITP occurs in a median of 15% of patients [9]. These patients are at lifelong risk for infection from *Streptococcus pneumoniae, Neisseria meningitides* and *Haemophilus influenza*[10].

Therefore, caution must be used in the treatment of ITP. Aggressive treatment should be reserved for patients with severe and symptomatic thrombocytopenia, and more treatment options are needed to further guide disease management. A successful treatment for any disease should be non-toxic, inexpensive and easily administered. The need for novel treatments for ITP is therefore imperative.

Here, we report two cases of patients with severe ITP who responded to high-dose vitamin D and Plaquenil® (hydroxychloroquine) but who failed conventional therapies.

## Case presentation

### Case 1

A 79-year-old Caucasian man was referred for management of severe ITP. Serology was positive for both anti-SSA/Ro autoantibodies (SSA) and antinuclear antibodies (ANA) suggesting that the patient had an overlap of systemic lupus erythematosus (SLE) and/or Sjögren’s syndrome. In addition, he was cytopenic and complained of dry eyes and mouth (sicca symptoms) and joint pain, which are secondary symptoms of SLE and/or Sjögren’s syndrome. The oncology clinic found no evidence of an underlying malignancy responsible for this life-threatening hematologic condition. The patient received WinRho® (IV Immune globulin, or IVIg) and high-dose prednisone (60mg/day) after his platelet count had dropped to 9000/mm^3^ (normal >140,000/mm^3^). Because the platelet count only increased to 43,000/mm^3^, the oncologist was concerned that prednisone alone was not effective. The patient was therefore referred to a rheumatology clinic for other therapeutic options.

To treat the ITP, we administered Plaquenil® (hydroxychloroquine) 200mg twice a day, prednisone 15mg/day and high-dose vitamin D 50,000IU once a week. The patient had a borderline low vitamin D level of 34ng/mL; in our experience with other autoimmune diseases, we have found an enhanced therapeutic response to standard treatment when a patient’s vitamin D level increases to 40ng/mL to 50ng/mL or above.

The patient’s platelet count increased slightly to 55,000/mm^3^ 2 weeks after this treatment began. To achieve an enhanced clinical benefit, vitamin D dosing was increased to 150,000IU per week and prednisone was lowered to 10mg/day; Plaquenil® (hydroxychloroquine) remained at 400mg/day.

His platelet count had increased to 114,000/mm^3^ 2 months after his initial visit. Prednisone was tapered to 5mg/day and vitamin D was lowered to 50,000IU twice a week whereas Plaquenil® (hydroxychloroquine) was not altered.

The patient’s platelet count had increased to 141,000/mm^3^ 3 months after the initial visit. Vitamin D was maintained at 100,000IU per week, prednisone was maintained at 5mg/day, and Plaquenil® (hydroxychloroquine) remained at 400mg/day.

The patient’s platelet count remained stable for approximately 6 months on this regimen. When his platelet counts stabilized (at 140,000/mm^3^), high-dose vitamin D supplementation was discontinued. He remained on 400mg/day Plaquenil® (hydroxychloroquine) and prednisone (5mg/day).

The patient’s platelet count dropped to 18,000/mm^3^ a month after vitamin D supplementation was discontinued. In response to this substantial decrease, the patient was again prescribed vitamin D at 50,000IU twice a week and prednisone 30mg/day; after 3 weeks, his platelet count had increased to 91,000/mm^3^.

A month later his platelet count increased to 137,000/mm^3^ and prednisone was reduced to 5mg/day (vitamin D and Plaquenil® (hydroxychloroquine) treatment did not change). The patient’s platelet count reached 161,000/mm^3^ 4 months later. With the patient clinically stable, prednisone treatment was discontinued. Vitamin D was reduced to 50,000IU once a week and Plaquenil® (hydroxychloroquine) was continued at the same dose as before.

His platelet count was 215,000/mm^3^ 6 weeks after discontinuing prednisone, indicating a regimen of Plaquenil® (hydroxychloroquine) 200mg twice a day and vitamin D 50,000IU once a week alone was sufficient to maintain a stable count. The patient remained stable off prednisone.

Even with high-dose vitamin D, there were no significant changes in calcium, phosphate, or creatinine levels.

### Case 2

An 87-year-old Caucasian woman was referred to our clinic from the oncology service with a platelet count of 8000/mm^3^. She presented with an ANA at 1:640, positive SSA, complained of dry eyes and dry mouth, and was cytopenic. Her total complement was also elevated at 86 hemolytic units. The patient was felt to have an overlap syndrome of SLE and Sjögren’s with an immune thrombocytopenia. Her initial 25-hydroxy (25-OH) vitamin D level was 17ng/mL.

She was started on Plaquenil® (hydroxychloroquine) 200mg twice a day and maintained on prednisone 40mg/day, which had been initiated by the hematology-oncology service. Because of severely low levels of 25-OH vitamin D, she was started on vitamin D 50,000IU once a week.

A month later the patient was doing well and vitamin D treatment was continued at 50,000IU once a week. Because her platelet count had increased to 72,000/mm^3^, her prednisone dose was tapered from 40mg to 15mg.

The patient’s platelet count had increased to 301,000/mm^3^ 6 weeks after her initial visit and she was therefore maintained on Plaquenil® (hydroxychloroquine), vitamin D (50,000IU once a week) and prednisone (tapered to 7.5mg).

Her platelet count dropped to 89,000/mm^3^ 6 weeks later. Her vitamin D treatment at 50,000IU was increased to twice weekly and prednisone and Plaquenil® (hydroxychloroquine) were maintained at 7.5mg and 400mg, respectively.

A month after starting the new high-dose vitamin D regimen the patient was doing well; she had no complaints and had a normal energy level. At this time, her platelet count had increased to 244,000/mm^3^ and therefore prednisone was tapered to 5mg. The patient’s vitamin D levels elevated to 89ng/mL. She continued on Plaquenil® (hydroxychloroquine; 400mg/day) and vitamin D 50,000IU twice a week.

After 9 weeks on this dosage (Plaquenil® (hydroxychloroquine) 400mg/day, vitamin D 100,000IU/week, and prednisone 5mg/day), her platelet count was 176,000/mm^3^, which increased to 212,000/mm^3^ a month later. At this time, the prednisone was decreased to 4mg/day and 6 weeks later her platelet count was 194,000/mm^3^.

She continued on prednisone 4mg/day and Plaquenil® (hydroxychloroquine) 400mg/day, and received a lower dose of vitamin D (50,000IU/week). After 16 weeks, her platelet count remained high at 182,000/mm^3^; prednisone was decreased to 2 mg/day.

The patient remained in remission 2.5 years after her initial presentation with a platelet count of 169,000/mm^3^ on vitamin D 50,000IU/month, prednisone 2mg/day and Plaquenil® (hydroxychloroquine) 400mg/day.

She remains stable 4.5 years post-initial presentation on a lower dose of Plaquenil® (hydroxychloroquine; 200mg/day) that was started 6 months prior, prednisone 2mg/day and vitamin D 50,000IU/month.

## Discussion

Immune thrombocytopenia is an autoimmune disease in which platelets are targeted by the host immune system. Platelet depletion occurs when autoantibodies targeting glycoproteins found on the surface of platelets opsonize the cells, resulting in destruction by macrophages. These antibodies can also bind to megakaryocytes and prevent complete maturation, which results in lowered levels of platelet production [11,12]. Several treatments are currently in use in the clinical setting that increase platelet counts, including steroid and immunosuppressive therapy.

Vitamin D, or 1,25-dihydroxyvitamin D3 (1,25(OH)2D3), may be a potent immunomodulator, and it may have potential therapeutic use in many autoimmune diseases. In 1991, researchers found that the production of interleukin (IL)-6 and IL-2 in cell cultures were reduced by 1,25(OH)2D3 [13]. It was postulated that 1,25(OH)2D3 may inhibit the production and function of IL-6 and, therefore, regulate lymphocyte functions. Activation of the vitamin D receptors (VDRs) is known to alter immune cell transcriptional patterns, proliferation and differentiation through several intracellular pathways [14]. Both CD4+ and CD8+ lymphocytes contain significant amounts of VDRs, and thus it is reasonable to conclude that 1,25(OH)2D3 may play a role in the regulation of the immune response [15].

It has been documented that patients with immune thrombocytopenia have high rates of mononuclear cell proliferation as well as lymphocytes that secrete greater amounts of IL-2 compared to controls [16]. Researchers have also shown that in these patients, depleting CD8+ lymphocytes and complement did not reduce proliferation of mononuclear cells, which indicates that CD4+ T helper cells may be responsible for the response [17]. This suggests that stimulated CD4+ T helper cells mediate antibody production and maturation of B lymphocytes, and may contribute to the eventual autoantibody response targeting these patients’ platelets.

Several studies have shown that 1,25(OH)2D3 can inhibit the development of multiple sclerosis in the murine model [18,19]. Researchers suggest that CD4+ T cells may be the target of 1,25(OH)2D3 immunosuppression and studies have found that 1,25(OH)2D3 can inhibit T-cell proliferation and activation. Other studies have shown that vitamin D stimulates transforming growth factor (TGF)-β1 and IL-4 production, which in turn may suppress inflammatory T-cell activity [20,21]. TGF-β1 may help direct T-cell differentiation and promote the production of T regulatory cells via the Foxp3 transcription regulatory pathway resulting in the observed immunosuppression [22]. In fact, studies have shown that TGF-β1 is able to promote T-cell differentiation from non-suppressor CD4+ T cells simply through Foxp3 induction [23]. These T regulatory cells help suppress the immune response and thus maintain immune system homeostasis and tolerance to self-antigens.

Although the number of T regulatory cells in ITP patients is significantly reduced [17], recent research has shown that it is possible to generate T regulatory cells *in vitro* by pharmacologically manipulating the dendritic cells with glucocorticoid and 1,25(OH)2D3 [24]. Drug-induced effects on dendritic cells included induction of IL-10-secreting regulatory T cells, attenuation of IL-2 and interferon gamma secretion, prevention of T-cell IL-4 secretion, and suppression of antigen-specific primary T-cell proliferation [14]. Overall, 1,25(OH)2D3 seems to have two important immunomodulatory functions: down-regulation of over proliferative CD4+ cells and concurrent up-regulation of T regulatory cells. Together, these effects may have the potential to reduce the autoantibody response and may potentially explain the restoration of platelet levels upon treatment of high-dose vitamin D in patients who have autoimmune thrombocytopenia.

## Conclusions

In this case report, we describe an association between vitamin D deficiency and immune thrombocytopenia in two patients. Both patients had SLE and/or Sjögren’s overlap with positive serology and profound life-threatening thrombocytopenia, all of which were unaffected by conventional therapies. High-dose replacement of vitamin D plus the addition of Plaquenil® (hydroxychloroquine) and modest doses of steroids resulted in normalization of both patients’ platelet counts. After discontinuation of supplemental vitamin D in one patient, the 25-OH vitamin D normalized, but the platelet count subsequently plummeted. Resuming vitamin D supplementation restored the platelet count to the previous normal range. We postulate that vitamin D may work synergistically with Plaquenil® (hydroxychloroquine) through unknown mechanisms; we have found the combined regimen works better than vitamin D alone. This treatment option may also down-regulate the proliferation of CD4+ cells and up-regulate T regulatory cells. These combined effects may potentially reduce the autoantibody response.

The successful treatment of ITP can be variable, and the toxicity of many of the existing treatments is considerable. The discovery and development of efficacious and non-toxic treatment for ITP is therefore imperative. Plaquenil® (hydroxychloroquine) has been used clinically for over 50 years and has an exceptional safety record. It is probably the safest among the disease-modifying agents that rheumatologists prescribe. Retinopathy is extremely rare, and the few reported cases were in patients taking the drug for more than 5 years. Baseline and annual eye exams are recommended [25]. Other less common side effects include rash, tinnitus and muscle aches. It should not be prescribed to patients with glucose-6-phosphate dehydrogenase deficiency.

The Case 1 patient was treated with prednisone in addition to vitamin D when platelet counts dropped subsequent to temporary vitamin D discontinuation. Although it is possible prednisone was responsible for the platelet count recovery at 3 months post-clinic admission, we feel it is unlikely for two reasons. First of all, the platelet count significantly decreased within one month of discontinuation of vitamin D. The dosage of prednisone remained unchanged during this period. Secondly, the patient’s platelet counts remained stable for 3 months without any prednisone treatment. We recommend that vitamin D levels be obtained on all patients who are thrombocytopenic; the high prevalence of vitamin D deficiency in the United States of America makes this particularly crucial. If vitamin D levels are less than 30ng/mL, we recommend the patient receive aggressive replacement with vitamin D supplementation at high doses.

In our experience the therapeutic dose varies between 50,000 and 100,000IU/week depending on the platelet count. This dose should be maintained for many months if the renal function remains normal. When the platelet count stabilizes, in some patients, vitamin D supplementation may need to be maintained indefinitely. We also suggest the addition of Plaquenil® (hydroxychloroquine) 200mg twice a day, especially if the patient is ANA positive and/or there is a clinical suggestion of Sjögren’s syndrome or SLE. We believe there is a synergistic effect of Plaquenil® (hydroxychloroquine) and high-dose vitamin D that is clinically important and thus recommend further studies be done to confirm our findings and elucidate the mechanism of this interaction. It is noteworthy that neither patient experienced any untoward side effect from high-dose vitamin D, suggesting it is a safe alternative treatment to a life-threatening condition.

## Consent

Written informed consent was obtained from the patients for publication of this manuscript and any accompanying images. A copy of the written consent is available for review by the Editor-in-Chief of this journal.

## Competing interests

The authors declare no competing interests.

## Authors’ contributions

BB is the patients’ physician. TBK aided in forming the discussion and conclusions. BB and TBK wrote the manuscript. All authors read and approved the final manuscript.
